# Impact of High-Dose Anti-Infective Agents on the Osteogenic Response of Mesenchymal Stem Cells

**DOI:** 10.3390/antibiotics10101257

**Published:** 2021-10-16

**Authors:** Jakob Hofmann, Sabrina Klingele, Uwe Haberkorn, Gerhard Schmidmaier, Tobias Grossner

**Affiliations:** 1 Clinic for Orthopedics and Trauma Surgery, Center for Orthopedics, Trauma Surgery and Paraplegiology, University Hospital Heidelberg, 69120 Heidelberg, Germany; jakob.hofmann@med.uni-heidelberg.de (J.H.); Gerhard.Schmidmaier@med.uni-heidelberg.de (G.S.); 2 Tissue & Cell Banking GmbH (TICEBA GmbH), 69120 Heidelberg, Germany; Sabrina.Klingele@ticeba.com; 3 Department of Nuclear Medicine, University Hospital Heidelberg, 69120 Heidelberg, Germany; uwe.haberkorn@med.uni-heidelberg.de; 4 Clinical Cooperation Unit Nuclear Medicine, Deutsches Krebsforschungszentrum (DKFZ), 69120 Heidelberg, Germany; 5 Translational Lung Research Center Heidelberg (TLRC), German Center for Lung Research (DZL), 69120 Heidelberg, Germany

**Keywords:** osteogenesis, anti-infective agents, ^99m^Tc-labeling, Gentamicin, Vancomycin, Voriconazole, nonunion, bone defect

## Abstract

Treatment of infected nonunions and severe bone infections is a huge challenge in modern orthopedics. Their treatment routinely includes the use of anti-infective agents. Although frequently used, little is known about their impact on the osteogenesis of mesenchymal stem cells. In a high- and low-dose set-up, this study evaluates the effects of the antibiotics Gentamicin and Vancomycin as well as the antifungal agent Voriconazole on the ability of mesenchymal stem cells to differentiate into osteoblast-like cells and synthesize hydroxyapatite in a monolayer cell culture. The osteogenic activity was assessed by measuring calcium and phosphate concentrations as well as alkaline phosphatase activity and osteocalcin concentration in the cell culture medium supernatant. The amount of hydroxyapatite was measured directly by radioactive ^99m^Technetium-HDP labeling. Regarding the osteogenic markers, it could be concluded that the osteogenesis was successful within the groups treated with osteogenic cell culture media. The results revealed that all anti-infective agents have a cytotoxic effect on mesenchymal stem cells, especially in higher concentrations, whereas the measured absolute amount of hydroxyapatite was independent of the anti-infective agent used. Normed to the number of cells it can therefore be concluded that the above-mentioned anti-infective agents actually have a positive effect on osteogenesis while high-dose Gentamycin, in particular, is apparently capable of boosting the deposition of minerals.

## 1. Introduction

The surgical therapy of critical size bone defects and bone nonunions presents a great challenge for orthopedic surgeons [1]. Especially, nonunions and defects caused by infections are extremely difficult to treat, sometimes taking months or even years to heal, if at all [2]. The treatment usually requires an aggressive regime of radical debridement of the bone combined with a long-term, high-dose antibiotic treatment [2]. Infections after fractions are not rare; their incidence ranges from 1% in low-energy closed fractures to over 30% in open tibia fractures [3,4], while high-risk patients are those with complex fractures and extensive tissue damage often leading to infected nonunions [5,6,7,8]. A general terminology for traumatic infections does not exist, but they are often referred to as infections after fracture fixation (IAFF) [9].

Besides traumatic injury, infection-related bone defects also occur in elective surgery. Total hip arthroplasty (THA) is one of the most performed procedures in orthopedics and is currently the only cure available for severe hip osteoarthritis [10]. Here, a rare but difficult to treat complication is the prosthetic joint infection (PJI) which occurs in up to 2.5% of all hip or knee arthroplasties [11,12] often resulting in a massive bone defect. Similar to the treatment of infection-related nonunions, treatment of PJIs include the intravenous administration of antibiotics as well as the debridement of the infection site [13]. Antibiotics are the essential backbone for the treatment of these bone defects.

Staphylococci are the bacteria most commonly isolated from infected nonunions and they contribute to over 50% of all bone infections [11,14]. Less frequent are Gram-negative bacilli, anaerobes, enterococci, and streptococci [14]. Infections are classified into early (<2 weeks), delayed and late (>10 weeks) [15]. Whereas early infections are typically caused by highly virulent *Staphylococcus aureus*, later infections can often be attributed to the less virulent *Staphylococcus epidermidis* [9]. These bacteria are capable of forming biofilms, which result in increased resistance to antibiotics [16]. Therefore, systemically administered antibiotics may have little to no effect on bacterial biofilms frequently found at infection sites [16]. To achieve much higher local concentrations of the antibiotics without harming the patient due to side effects, antibiotic-loaded bone cement can be inserted into the defect after radical surgical debridement, leading to significantly higher concentrations of the agent [17,18]. In these bone cements, Gentamicin is one of the most used antibiotics, mostly in combination with the glycopeptide Vancomycin [19,20]. However, it seems that high concentrations of Gentamicin may be cytotoxic [21], although findings are inconsistent and the effects of Gentamicin on osteogenesis are highly contested.

Antibiotic resistance is increasing in bacteria that cause bone infections [22]. *Staphylococcus aureus* causes around 30% of all bone infections, however, over 50% of the bacteria are resistant to methicillin (MRSA) [23]. Bone infections with MRSA are typically treated with Vancomycin [24,25,26]. Here, Vancomycin can be either administered intravenously or locally by means of antibiotic-loaded cement spacers [27] but little to nothing is known about the effects of Vancomycin on osteogenesis.

Less prevalent are infections with fungi, while, in the clinical setting, these infections are often disastrous for the patient and very complicated to treat [28]. Like bacteria, fungi are capable of forming biofilms [29]. This makes a fungal implant infection much harder to treat, as fungi organized in biofilms require up to 1000 times the concentration of antifungal agents compared to planktonic fungi [30]. For this reason, local administration of antifungals by antifungal-loaded bone cement (ALBC) is the preferred form of treatment [31]. In ALBCs, the azole Voriconazole is one of the most frequently used antifungal agents [32] but again, no data are available concerning its potential effect on the local osteogenic response.

As the findings regarding the effects of anti-infective agents on osteogenesis and osteoblast activity are highly inconsistent, this study was performed to reveal new insights which could potentially be very important for enabling clinicians and fundamental researchers to acquire a better understanding of the treatment of bone infections.

Within the study design, the effects of low-dose concentration (systemic administration of the drugs) and high-dose concentration (local administration of the drugs) of Gentamycin, Vancomycin, and Voriconazole on the osteogenic differentiation potential of human mesenchymal stem cells were investigated using the novel and highly sensitive method ^99m^Tc-HDP labeling. This is a non-destructive method to quantify the amount of hydroxyapatite produced in monolayer or 3D-cell cultures [33].

Our results revealed that there is a dose-dependent negative effect for all anti-infective drugs upon hMSC cell proliferation. However, the remaining cells showed a higher osteogenic differentiation potential, when treated with any anti-infective drugs, compared to the standard osteogenic differentiation assay—especially for high-dose Gentamycin.

## 2. Results

### 2.1. DAPI Cell Count

**Figure 1:**
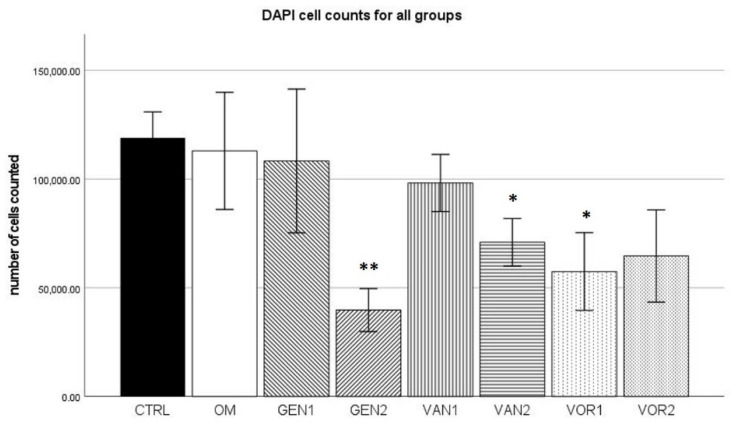
Mean cell count per group as established by the means of DAPI cell count. The error bars show the error of mean. Significances are indicated by stars: bold stars indicate a significance compared to the negative control CTRL, stars in italics indicate a significance compare to the osteogenic control OM. * = significance *p* ≤ 0.05, ** = significance *p* ≤ 0.01.

The negative control group (no osteogenic differentiation) showed the highest mean (118,932 cells) out of all groups. The osteogenic differentiation without any anti-infective agents (OM) had a mean of 112,983 cells per sample. Group GEN2 showed the lowest value with a mean of 39,754 cells per sample (see Figure 1).

According to the Kolmogorov–Smirnov test, all groups showed a normal distribution of the number of cells measured. In the Mann–Whitney U test, groups GEN2, VAN2, and VOR1 showed a significantly lower cell count compared to the control group CTRL. In all three cases, the effect power was high (*r* > 0.5).

### 2.2. Calcium Concentration in Cell Culture Medium

**Figure 2:**
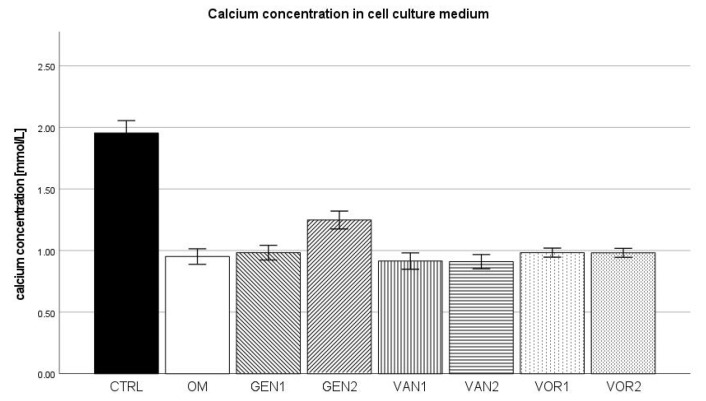
Mean calcium concentration per group as measured in the cell culture medium. The error bars show the error of mean.

In the negative control group, a mean calcium concentration of 1.955 mmol/L was measured. In the osteogenic control group, the mean calcium concentration was 0.952 mmol/L. The highest calcium concentration was measured in the negative control group. Out of all osteogenic groups, group GEN2 showed the highest calcium concentration, whereas group VAN2 showed the lowest calcium concentration (see Figure 2).

**Figure 3:**
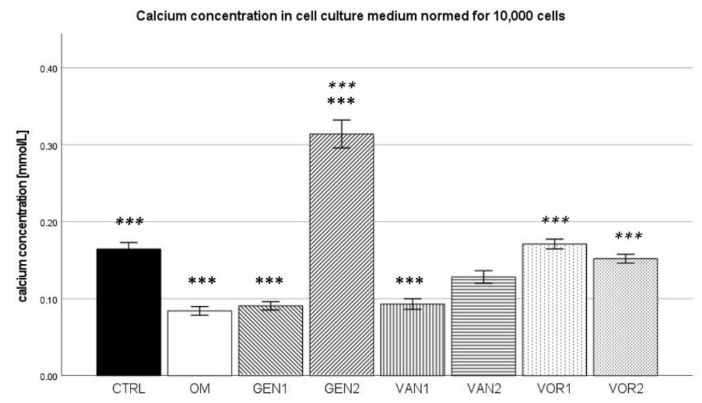
Mean calcium concentration per group as measured in the cell culture medium, normed for 10,000 cells. The error bars show the error of mean. Significances are indicated by stars: bold stars indicate a significance compared to the negative control CTRL, stars in italics indicate a significance compare to the osteogenic control OM. *** = significance *p* ≤ 0.001.

The results were then normed for 10,000 cells and further analysis was performed (see Figure 3). In the Kolmogorov–Smirnov test, all groups showed normal distribution, except group VAN2, which was subsequently excluded in the ANOVA analysis. In the Bonferroni post-hoc test, groups OM, GEN1, GEN2, and VAN1 showed a calcium concentration significantly different from the negative control group (*p* < 0.001). Compared to the osteogenic control group, groups GEN2, VOR1, and VOR2 showed a significantly higher calcium concentration (*p* < 0.001).

### 2.3. Phosphate Concentration in Cell Culture Medium

**Figure 4:**
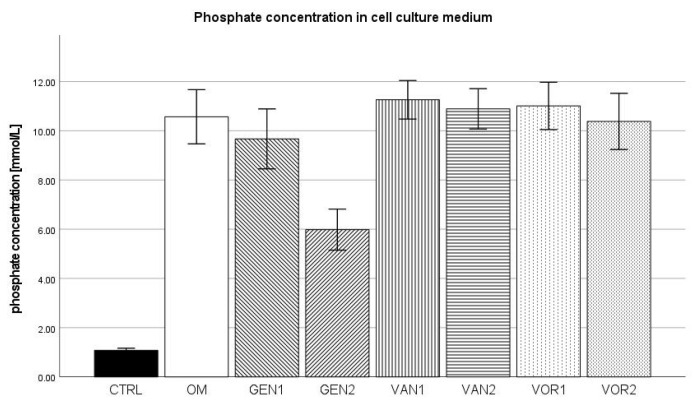
Absolute mean phosphate concentration per group as measured in the cell culture medium. The error bars show the error of mean.

The negative control showed a mean phosphate concentration of 1.082 mmol/L and the osteogenic control group 10.572 mmol/L. The negative control group showed the lowest phosphate concentration amongst all groups, whereas group VOR1 showed the highest with 11.012 mmol/L. Out of all osteogenic groups, group GEN2 showed the lowest phosphate concentration with 5.985 mmol/L (see Figure 4).

**Figure 5:**
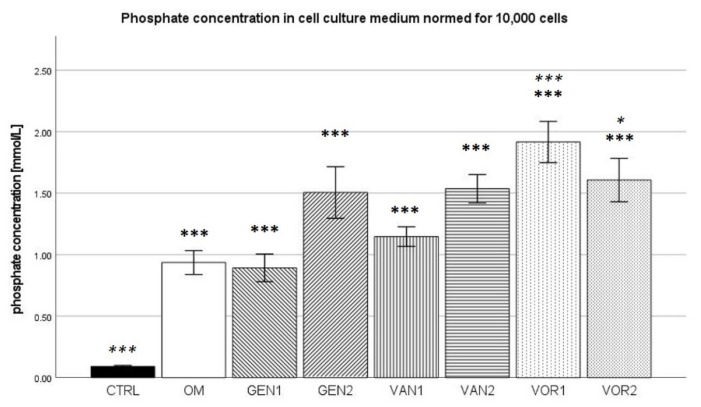
Mean phosphate concentration per group as measured in the cell culture medium, normed for 10,000 cells. The error bars show the error of mean. Significances are indicated by stars: bold stars indicate a significance compared to the negative control CTRL, stars in italics indicate a significance compare to the osteogenic control OM. * = significance *p* ≤ 0.05, *** = significance *p* ≤ 0.001.

The results were then normed for 10,000 cells and further analysis was performed (see Figure 5). In the Kolmogorov–Smirnov test, results for all groups showed a normal distribution. In the ANOVA with Bonferroni post-hoc test, all groups presented a significantly higher phosphate concentration compared to the negative control (*p* < 0.001). The osteogenic control group was only significantly different from groups VOR1 (*p* < 0.001) and VOR2 (*p*< 0.05), which showed higher phosphate concentrations.

### 2.4. Osteocalcin Concentration in Cell Culture Medium

**Figure 6:**
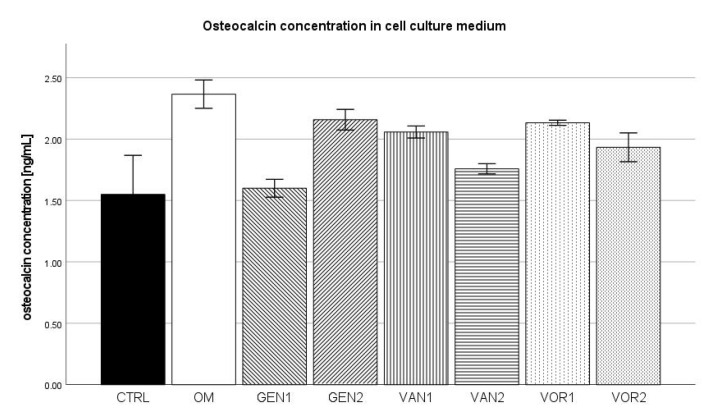
Mean osteocalcin concentration per group as measured in the cell culture medium. The error bars show the error of mean.

The negative control group showed a mean osteocalcin concentration of 1.55 ng/mL while the osteogenic control group showed a mean osteocalcin concentration of 2.37 ng/mL. This was the highest concentration among all groups, whereas the negative control showed the lowest concentration among all groups. Out of all osteogenic groups, GEN1 showed the lowest osteocalcin concentration with 1.60 ng/mL (see Figure 6).

**Figure 7:**
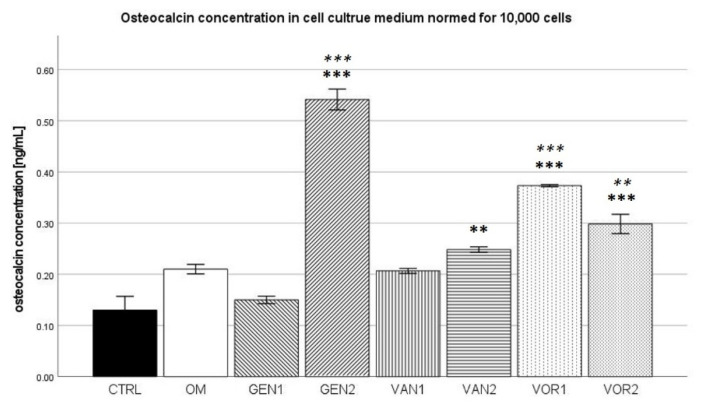
Mean osteocalcin concentration per group as measured in the cell culture medium, normed for 10,000 cells. The error bars show the error of mean. Significances are indicated by stars: bold stars indicate a significance compared to the negative control CTRL, stars in italics indicate a significance compare to the osteogenic control OM. ** = significance *p* ≤ 0.01, *** = significance *p* ≤ 0.001.

The results were then normed for 10,000 cells and further analysis was performed (see Figure 7). In the Kolmogorov–Smirnov test, two groups did not show a normal distribution, CTRL VOR1. VOR1 did not show a normal distribution because all measurements were nearly identical, therefore the group was still included in the Bonferroni post-hoc test. CTRL did not show a normal distribution, because one measurement substantially differed from the mean (3.1 ng/mL as compared to 1.55 ng/mL). When this one measurement was not regarded, group CTRL showed a normal distribution.

In the Bonferroni post-hoc test, groups GEN2, VOR1 and VOR2 showed a significantly higher osteocalcin concentration compared to the negative CTRL (*p* < 0.001). Group VAN2 also showed a significantly higher osteocalcin concentration compared to group CTRL (*p* < 0.01). Compared to the osteogenic control OM, groups GEN2 and VOR1 showed a significantly higher osteocalcin concentration (*p* < 0.001), as well as group VOR2 (*p* < 0.01).

### 2.5. Alkaline Phosphatase Activity in Cell Culture Medium

**Figure 8:**
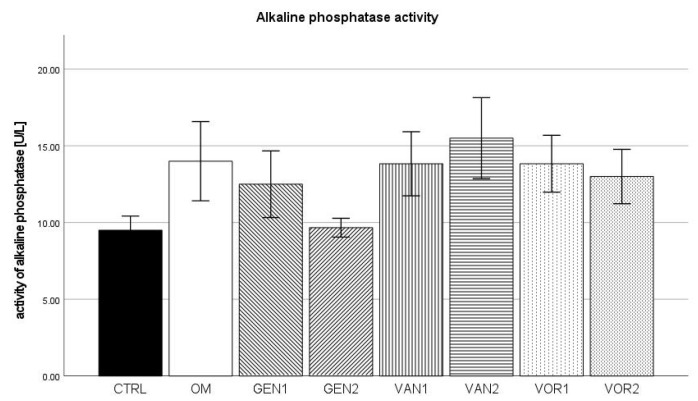
Mean AP activity per group as measured in the cell culture medium. The error bars show the error of mean.

The negative control group showed a mean alkaline phosphatase (AP) activity of 9.5 U/L and the osteogenic control group 14 U/L. The highest activity was measured in group VAN2 with 15.5 U/L. Out of all osteogenic groups, GEN2 showed the lowest activity with 9.7 U/L (see Figure 8).

**Figure 9:**
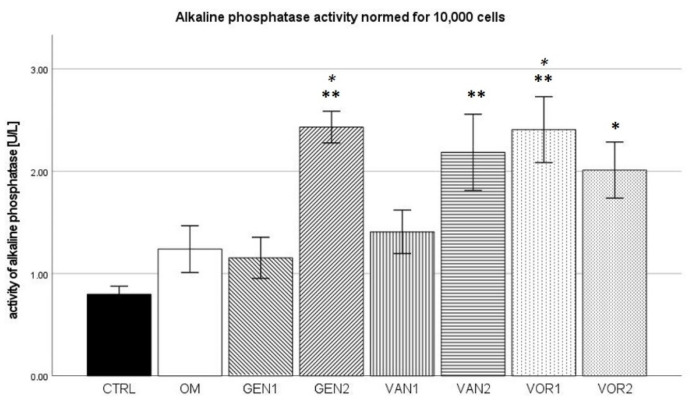
Mean AP activity per group as measured in the cell culture medium, normed for 10,000 cells. The error bars show the error of mean. Significances are indicated by stars: bold stars indicate a significance compared to the negative control CTRL, stars in italics indicate a significance compare to the osteogenic control OM. * = significance *p* ≤ 0.05, ** = significance *p* ≤ 0.01.

The results were then normed for 10,000 cells and further analysis was performed (see Figure 9). In the Kolmogorov–Smirnov test, all groups showed a normal distribution. In the Bonferroni post-hoc test, compared to the negative control, groups GEN2, VAN2, and VOR1 showed a significantly higher AP activity (*p* < 0.01), as well as group VOR2 (*p* < 0.05). Compared to the osteogenic control, groups GEN2 and VOR1 showed a significantly higher AP activity (*p* < 0.05).

### 2.6. ^99m^Technetium Labeling (Activimeter)

**Figure 10:**
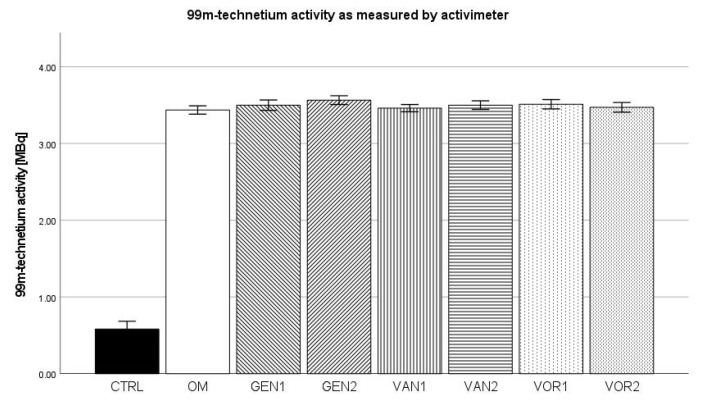
Mean ^99m^technetium uptake per group as measured by the activimeter. The error bars show the error of mean.

The negative control group showed a mean activity of 0.580 MBq. The osteogenic control showed a mean activity of 3.44 MBq, which was the lowest amongst all osteogenic groups. The highest uptake of 3.57 MBq was shown by group GEN2 (see Figure 10).

**Figure 11:**
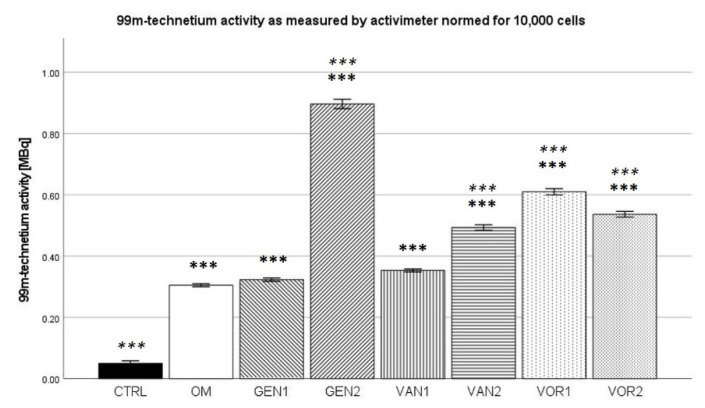
Mean ^99m^technetium uptake per group as measured by activimeter, normed for 10,000 cells. The error bars show the error of mean. Significances are indicated by stars: bold stars indicate a significance compared to the negative control CTRL, stars in italics indicate a significance compare to the osteogenic control OM. *** = significance *p* ≤ 0.001.

The results were then normed for 10,000 cells and further analysis was performed (see Figure 11). In the Kolmogorov–Smirnov test, all groups showed a normal distribution. Compared to the negative control group, all osteogenic groups showed a significantly higher uptake (*p* < 0.001). Compared to the osteogenic control (OM), groups GEN1 and VAN1 were not significantly different. All other osteogenic groups showed higher normed activity than the osteogenic control (*p* < 0.001).

### 2.7. ^99m^Technetium Labeling (Gamma Camera)

**Figure 12:**
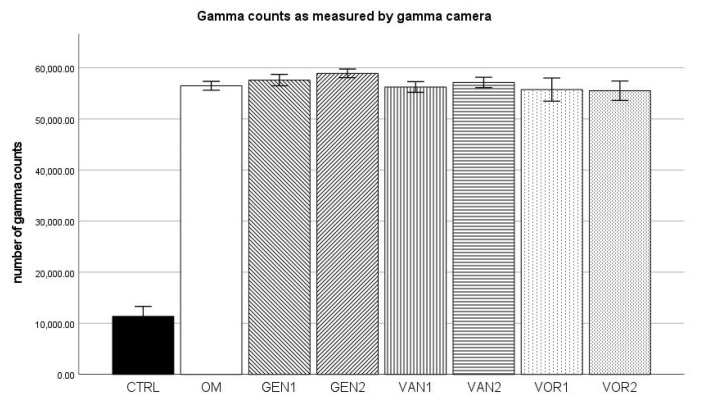
Mean gamma counts per group as measured by the gamma camera. The error bars show the error of mean.

For the negative control group, a mean of 11,358 gamma counts per second was measured and for the osteogenic control group a mean of 56,496 gamma counts per second. Group GEN2 showed the highest gamma counts with 58,924 per second, VOR2 showed the lowest gamma counts with 55,528 per second (see Figure 12).

**Figure 13:**
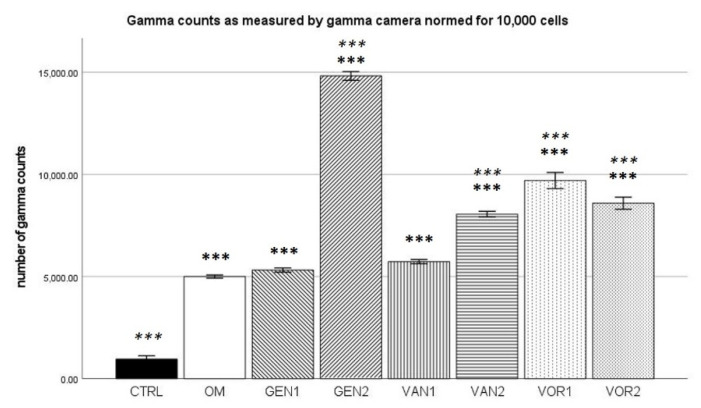
Mean gamma counts per group as measured by the gamma camera, normed for 10,000 cells. The error bars show the error of mean. Significances are indicated by stars: bold stars indicate a significance compared to the negative control CTRL, stars in italics indicate a significance compare to the osteogenic control OM. *** = significance *p* ≤ 0.001.

The results were then normed for 10,000 cells and further analysis was performed (see Figure 13). All measured values showed a normal distribution in the Kolmogorov–Smirnov test. In the Bonferroni post-hoc test, all osteogenic groups present a significantly higher number of gamma counts per second compared to the negative control group (*p* < 0.001). Except for groups GEN1 and VAN1, all osteogenic groups showed a significantly higher number of gamma counts per second compared to the osteogenic control group (*p* < 0.001).

## 3. Discussion

The DAPI cell count revealed, that the anti-infective agents have significant cytotoxic properties. All anti-infective agents showed a trend to a reduction in the number of cells in culture, although only the results for high-dose Gentamicin, high-dose Vancomycin, and low-dose Voriconazole were statistically significant. These findings are known and concur with studies examining the adverse effects of anti-infective agents. In a study by Rathbone et al., Gentamicin concentration of 100 µ/mL already led to a reduction in the cell count of 26–49% [34]. For Vancomycin the effect is less pronounced. Still, Antoci et al. were able to show that Vancomycin concentrations of 125 µg/mL and upward have a negative effect on cell numbers, leading to a substantial reduction of the number of cells counted [35]. For Voriconazole, a number of adverse effects are described in the literature, among them osteomyelitis, caused by the release of fluoride, which is part of the chemical structure of Voriconazole [36]. Fluoride is known to be toxic to the bone in high concentrations [37]. Schmidt et al. have demonstrated that Voriconazole concentrations of 100 µg/mL were capable of inhibiting osteoblast growth. Concentrations of 1000 µg/mL and above inhibited cell growth permanently, even after exposure stopped [28]. Such high doses, however, are untypical of clinical practice where concentrations achieved by drug-eluting spacers or systemic therapy are much lower [38,39], which is why we did not evaluate the effect of these extremely high doses. In our study, both Voriconazole concentrations (2 and 5 µg/mL) resulted in a lower cell count, although both concentrations were significantly lower than the concentrations described by Schmidt et al.

The fact, that Gentamicin, Vancomycin, and Voriconazole are cytotoxic requires that all results assessing the bone-formation capabilities of human mesenchymal stem cells under the influence of these anti-infective agents are normed to each cell count. Otherwise, it cannot be determined later whether a change in the formation of hydroxyapatite is due to a reduced number of cells or rather due to a reduced metabolic rate of the osteoblast-like cells. Similarly, it may be that absolute osteogenesis does not change while the individual metabolic rate of the surviving cells is drastically altered.

This is immediately clear when looking at the calcium concentration in the cell culture medium. The absolute concentration of calcium differs little between all groups except the negative control group CTRL, where the calcium concentration is substantially higher. This can be seen as inverse proof of the successful osteogenic differentiation of all osteogenic groups, as a reduction of calcium in the cell culture medium is known to be an inverse noninvasive marker for osteogenesis [40]. When normed for 10,000 cells, groups with a lower cell count show a higher calcium concentration. Groups GEN2, VOR1, and VOR 2 show a significantly higher calcium concentration compared to the osteogenic control, which did not receive any anti-infective agents.

The absolute concentrations for phosphate showed a substantially lower concentration for the negative control group. This can probably be considered as proof for successful osteogenesis within the osteogenic groups, although little is known about the role of phosphate as a marker for osteogenesis. When normed for 10,000 cells, groups with a lower cell count again show higher phosphate concentrations, and the groups VOR1 and VOR2 are significantly different from the osteogenic control.

The absolute concentration for osteocalcin was the lowest in the negative control group and the highest in the osteogenic control group. It is known that secretory osteocalcin can be used as a marker for osteogenesis [41]. The low concentration in the negative control and the high concentration in the osteogenic control can thus be seen as proof for successful osteogenic differentiation of the mesenchymal stem cells. In the osteogenic groups treated with anti-infective agents, osteocalcin concentration is also higher than in the negative control, but reduced compared to the osteogenic control, showing the inhibitory influence of these anti-infective agents on osteogenesis.

In the absolute measurement, alkaline phosphatase showed little difference between the groups. This is not surprising, as alkaline phosphatase is only an indirect method to establish the osteogenic activity of cells and can as such be subject to a number of effects [42]. In the normed analysis, groups GEN2, and VOR1 are significantly different from the osteogenic control OM. However, this is probably due to the significantly reduced number of cells in these groups.

The absolute measurement of hydroxyapatite formation through labeling with ^99m^Tc showed that in all osteogenic groups there was both a significantly higher amount of ^99m^Tc activity and gamma counts compared to group CTRL (nonosteogenic negative control group). This is well known as proof for successful osteogenic differentiation of the mesenchymal stem cells.

**Figure 14:**
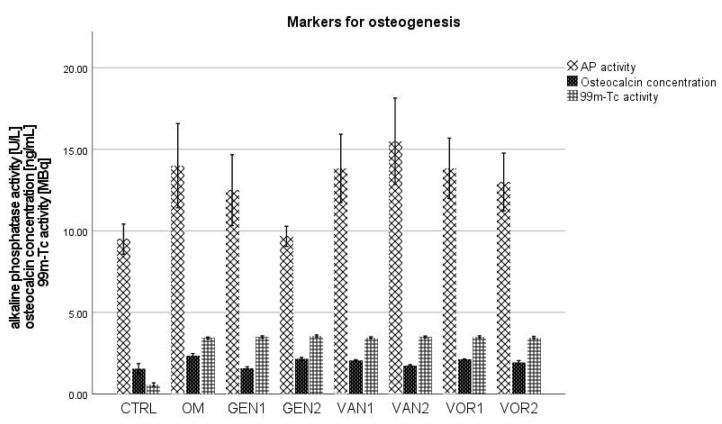
Mean values for the osteogenic markers AP activity, osteocalcin concentration and ^99m^Technetium activity per group. The error bars show the error of mean.

When considering the osteocalcin concentration, alkaline phosphatase activity, and the ^99m^Technetium activity, it seems that the osteogenic differentiation of the mesenchymal stem cells was successful. All of these markers were lowest in the negative control group and substantially higher in the osteogenic groups (see Figure 14).

Among the osteogenic group, the absolute measurement of ^99m^Tc activity and gamma counts did not show any difference between the groups. The overall osteogenic potential does not seem to be influenced by any of the anti-infective agents used. However, when normed for 10,000 cells, a number of effects can be seen. Compared to the osteogenic control, the relative formation of hydroxyapatite is greatly improved in the group GEN2. This finding is contrary to a study by Isefuku et al., which showed that Gentamicin has a negative effect on osteogenesis [21]. However, here the results were not normed to the number of cells, which is a clear limitation of that study. Only the effect on absolute osteogenesis was described. Additionally, a different study by Rao et al. was able to show that another aminoglycoside, Tobramycin, does improve osteogenic differentiation and is therefore beneficial to bone healing [43]. According to the assessment of hydroxyapatite using technetium labeling, it seems that Gentamicin has a positive effect on osteogenic differentiation. The uptake normed for 10,000 cells for Gentamicin in high concentrations was higher than in all other groups. Although Gentamicin is cytotoxic and fewer cells remain, the remaining cells demonstrate an increased ability to form hydroxyapatite.

Group VAN2 also showed a significantly higher ^99m^Tc activity and gamma counts compared to the osteogenic control when normed for 10,000 cells, meaning a much higher relative hydroxyapatite formation by the individual cell. In the existing literature, the exact effects of Vancomycin on bone formation are highly contested. While Booysen et al. suggest in their 2018 study that Vancomycin has no effect whatsoever on mesenchymal stem cell viability and osteogenesis, even at high doses [44], an in vitro study by Eder et al. shows that Vancomycin might interfere with bone regeneration [45]. This may be due to the decline in pH after the administration of Vancomycin at an infection site. Low pH levels are known to induce cell death in osteoblasts [46]. This fact concurs with our findings, as the DAPI cell count showed that Vancomycin is capable of significantly reducing the cell count, and this effect increases with higher concentrations. However, the technetium labeling revealed no difference between the absolute hydroxyapatite formation in the Vancomycin groups and the osteogenic control. It can therefore be concluded, that although Vancomycin is toxic for osteoblasts, especially in high concentrations, the remaining cells show an increased capability of forming hydroxyapatite.

For Voriconazole, both groups (VOR1 and VOR2) were significantly different from the osteogenic control when normed for 10,000 cells, each of them showing a greater relative ability to form hydroxyapatite. This concurs with other findings. Allen et al. report that Voriconazole enhances osteoblast proliferation and osteoblastic activity as well as osteoblast differentiation [32]. We found, that although the cell count was lower compared to the negative control, individual osteogenesis was enhanced, so that overall osteogenesis did not differ from the control differentiation without the addition of anti-infective agents.

This means that although cytotoxic effects do take place with all used anti-infective agents, the remaining cells are capable of compensating for the loss of osteogenic potential. Exactly how this happens is not yet understood.

In clinical practice, the use of anti-infective agents might even be clearly beneficial in some cases. Bone marrow mesenchymal stem cells (bmMSC) are capable of detecting bacterial structures via toll-like receptors (TLR) [47]. A well-known effect of TLR ligation in bmMSC is the increased secretion of the pro-inflammatory cytokines IL-6 and IL-8 [48]. While the effects of IL-8 on osteogenesis are not yet clearly understood, IL-6 seems to be antiestrogenic by acting pro-osteoclastogenic and modulating bone homeostasis [49]. Thus, the removal of bacteria using antibiotics might further improve osteogenesis by suppressing these inflammatory pathways.

A limitation of our study is that all experiments were performed in vitro and therefore the results cannot be directly transferred into clinical practice. Not all mechanisms may be exactly the same in the living organism, especially in terms of the liver first pass and enzyme modification on the drugs. However, our results can serve as the basis for further in vivo studies for subsequent investigations into this very important aspect of septic bone surgery to gain more insights so that a better patient outcome can be achieved in the future. Still, for now, it appears that the treatment of bone infections using the anti-infective agents Gentamicin, Vancomycin, and Voriconazole might have cytotoxic effects that lead to a reduced number of mesenchymal stem cells capable of regenerating the bone defect. However, due to mechanisms that still need to be determined, the remaining cells show an increased ability to form hydroxyapatite, so that overall osteogenesis is not negatively affected, regardless of the anti-infective agent and concentration used.

## 4. Materials and Methods

### 4.1. Experimental Design at a Glance

Human mesenchymal stem cells were harvested from the femoral bone cavity of healthy donors (*n* = 6). A total of 2.5 × 10^5^ cells of each donor were seeded into T-150 flat bottom flasks and cultivated in expansion medium containing DMEM LG, 10% FCS, and 1% Penicillin/Streptomycin. After 5 days of expansion, cells from each donor were transferred into eight 35 mm diameter Petri dishes. Dish 1 was treated as a negative control (CTRL) and subsequently received DMEM low glucose 10% FCS, and 1% Penicillin/Streptomycin as a media. Dishes 2–8 were differentiated towards the osteogenic lineage using the standard osteogenic supplements dexamethasone (end concentration 100 nM), ascorbic acid (end concentration 50 µM), and ß-glycerol phosphate (end concentration 10 nM) using DMEM low glucose with 10% FCS but no Penicillin/Streptomycin as basal cell culture media. Each of the dishes 3–8 received a specific dose of an anti-infective agent: 8 µg/mL Gentamicin (Dish 3), 60 µg/mL Gentamicin (Dish 4), 20 µg/mL Vancomycin (Dish 5), 150 µg/mL Vancomycin (Dish 6), 2 µg/mL Voriconazole (Dish 7), and 5 µg/mL Voriconazole (Dish 8).

1. Group CTRL: Cell culture with DMEM low glucose containing 10% FCS and 1% Penicillin/Streptomycin.

2. Group OM: Osteogenic differentiation with DMEM low glucose containing 10% FCS and osteogenic supplements (100 nM dexamethasone, 50 µM ascorbic acid, 10 nM ß-glycerol phosphate).

3. Group GEN1: Osteogenic differentiation with DMEM low glucose containing 10% FCS and osteogenic supplements (100 nM dexamethasone, 50 µM ascorbic acid, 10 nM ß-glycerol phosphate), and Gentamicin (8 µg/mL).

4. Group GEN2: Osteogenic differentiation with DMEM low glucose containing 10% FCS and osteogenic supplements (100 nM dexamethasone, 50 µM ascorbic acid, 10 nM ß-glycerol phosphate), and Gentamicin (60 µg/mL).

5. Group VAN1: Osteogenic differentiation with DMEM low glucose containing 10% FCS and osteogenic supplements (100 nM dexamethasone, 50 µM ascorbic acid, 10 nM ß-glycerol phosphate), and Vancomycin (20 µg/mL).

6. Group VAN2: Osteogenic differentiation with DMEM low glucose containing 10% FCS and osteogenic supplements (100 nM dexamethasone, 50 µM ascorbic acid, 10 nM ß-glycerol phosphate), and Vancomycin (150 µg/mL).

7. Group VOR1: Osteogenic differentiation with DMEM low glucose containing 10% FCS and osteogenic supplements (100 nM dexamethasone, 50 µM ascorbic acid, 10 nM ß-glycerol phosphate), and Voriconazole (2 µg/mL).

8. Group VOR2: Osteogenic differentiation with DMEM low glucose containing 10% FCS and osteogenic supplements (100 nM dexamethasone, 50 µM ascorbic acid, 10 nM ß-glycerol phosphate), and Voriconazole (5 µg/mL).

Media change was every two days. After 21 days, the cell culture was terminated, and the mineral content was accessed. Samples for the analyses of the supernatant were collected on days 7, 14, and 21.

### 4.2. Harvest of HMSC

Bone marrow aspirates were obtained from the proximal femoral cavity of six healthy donors (*n* = 6) under general anesthesia during an elective surgical procedure for total hip arthroplasty after informed consent. During preparation of the proximal femoral bone cavity, 10 mL of bone marrow was collected into a 20 mL syringe (BD, Heidelberg, Germany) containing 1000 IU of heparin (Braun, Melsungen, Germany). Individual samples were diluted 1:1 with PBS (Gibco, Frankfurt, Germany) and washed twice with PBS. The mononuclear cell fraction was isolated by Ficoll gradient centrifugation (Ficoll-Paque-PLUS; Cytiva, Freiburg, Germany). Mononuclear cells were plated in T-150 polystyrene tissue culture flasks (Falcon, Kaiserslautern, Germany) at a density of 5·10^5^/cm^2^ and cultured in a humidified 5% CO_2_ atmosphere at 37 °C in high-glucose Dulbecco’s modified Eagle’s medium (DMEM-HG, Gibco, Germany) containing 10% heat-inactivated (56 °C, 30 min) fetal bovine serum (Sigma, Schnelldorf, Germany) and 1% Penicillin/Streptomycin (Sigma). After 48 h, all nonadherent cells were removed by washing with PBS while the adherent cells were defined as bone marrow mesenchymal stem cells. Media was changed three times a week (every 2–3 days). At 90% of confluence, cells were trypsinized (Trypsin-EDTA, Sigma) and stored as P0 cells for further experiments in liquid nitrogen. Each 0.5 mL Aliquot contained 5·10^5^ cells in 10% DMSO (Sigma).

### 4.3. HMSC Expansion

P0 human MSCs (*n* = 6) were thawed and seeded into T-150 flasks (Falcon) with 250,000 cells each and cultured for 10 days in a humidified 5% CO_2_ atmosphere at 37 °C. Media was changed every 2 days. The expansion media used was HG-DMEM with 10% FCS and 1% Pen/Strep. After 5 days, 80–90% confluence was reached in all cell cultures, and cells were trypsinized.

### 4.4. HMSC Differentiation

Cells from every donor (*n* = 6) were seeded in duplicates into eight different 35-mm flat bottom Petri dishes (Corning, Kaiserslautern) at a density of 10,000 cells/cm^2^, reflecting 8 groups per donor. Each of the dishes received its corresponding differentiation media (see 4.1. experiment at a glance). One group was treated as a negative control and did not receive any osteogenic supplements nor anti-infective agents. Another group was treated as an osteogenic control and did not receive any anti-infective agents. The anti-infective agents used were Gentamicin (Gentamicin-ratiopharm, Ratiopharm, Ulm, Germany), Vancomycin (Vanco-ratiopharm, Ratiopharm) and Voriconazole (V-Fend, Pfizer, Karlsruhe, Germany).

According to studies examining the therapeutical drug concentration in the blood serum, the following serum level concentrations were chosen: 8 µg/mL for Gentamicin [50], 20 µg/mL for Vancomycin [51] and 2 µg/mL for Voriconazole [38]. The anti-infective agent concentrations achieved by drug-eluting spacers were chosen according to studies examining the pharmacokinetics of spacers: 60 µg/mL for Gentamicin [19,52], 150 µg/mL for Vancomycin [19,52] and 5 µg/mL for Voriconazole [39].

Cells were cultures for 21 days in a humidified 5% CO_2_ atmosphere at 37 °C with media change three times a week (every 2–3 days). After 7, 14, and 21 days, the supernatant cell culture media was collected and stored in 1 mL Aliquots at −20 °C for further analysis. After 21 days the cell cultures were terminated. For ^99m^Tc-HDP labeling, they were washed twice with PBS followed by air drying under a cell culture hood. For the DAPI cell count, cell cultures were directly fixated with paraformaldehyde as described below.

### 4.5. DAPI Cell Count

After termination of the cell culture, one of the two duplicates in each group was incubated with 4% paraformaldehyde (PFA, Sigma) at room temperature for 20 min followed by fixation with 0.1% PFA. Subsequently, the fixated samples were stained with DAPI. Cells were counted under a microscope (Leica DMi8, Leica, Wetzlar, Germany). For each sample, 5 vision fields were selected at random and the cells were counted within these. The counts were assessed for normal distribution using the Kolmogorov–Smirnov test (see also 4.8) and their mean was taken to norm for 10,000 cells during the ^99m^Tc-HDP analysis and analysis of cell culture medium. Regarding the specifications of the microscope, each vision field had an area of 265,172 µm^2^. Knowing the area of the entire corning dish, the absolute number of cells in the sample was calculated.

### 4.6. ^99m^Tc-HDP Labeling and Analysis

A tracer aliquot of 5.3 MBq ^99m^Tc-HDP in 1 mL of NaCl 0.9% was added to each dish. The technetium activity was assayed with a dose calibrator (Activimeter ISOMED 1010, Nuklear-Medizintechnik Dresden GmbH, Dresden, Germany). The dishes were then incubated at room temperature for 2 h. The remaining liquid ^99m^Tc-HDP was removed and the dishes were washed twice in PBS to remove the unbound radiotracer. The dishes were then analyzed first with the dose calibrator to precisely determine the amount of bound tracer followed by imaging with a gamma camera (E.CAM+, Siemens, Erlangen, Germany). The dishes were placed directly on the lower detector and the counts were acquired for 180 s using a 256 × 256 detection grid. The images were analyzed using Xeleris Software (GE Healthcare, Frankfurt, Germany). Here, the total number of counts for each culture dish was determined by placing a circular region of interest (ROI) around each dish. All ROIs had the same pixel size and were chosen a bit smaller than the true diameter of the dish to avoid a partial volume effect. The software calculated the total gamma counts emitted from each ROI over the 180 s.

For each group, *n* = 6 samples were measured once in the activimeter and once under the gamma camera. Results were normed for 10,000 cells.

### 4.7. Analyzation of Cell Culture Media

The supernatant media which were collected after 7, 14, and 21 days were thawed and analyzed by the central laboratory of the University Hospital Heidelberg using a Siemens Dimension EXL200 (Siemens AG, Erlangen, Germany). Here, the calcium concentration, phosphate concentration, osteocalcin concentration, and the activity of alkaline phosphatase were measured using validated methods for analyzing cell culture media.

The analyzation of calcium concentration, phosphate concentration, osteocalcin concentration and the activity of alkaline phosphatase, respectively, was performed once per group using *n* = 6 samples per group and analyzation (calcium concentration, phosphate concentration, osteocalcin concentration activity of alkaline phosphatase). Results were normed for 10,000 cells.

### 4.8. Statistics

All results were tested for normal distribution using the Kolmogorov–Smirnov test before further tests were performed. For the DAPI cell count, a Mann–Whitney U test was then performed comparing all groups to the control group. For all other cases, an ANOVA followed by post-hoc test by Bonferroni was performed to determine statistical significances between the groups.

Since a significant reduction of cells was observed for some groups in the DAPI cell count, the results were normed for 10,000 cells.

Statistical analyses were performed using SPSS Statistics^®^ (IBM, Armonk, NY, USA) Version 27. Statistical significance was set to *p* ≤ 0.05.

## Data Availability

Supplementary data can be obtained upon request from the authors.
